# Interaction between PD-L1 and soluble VEGFR1 in glioblastoma-educated macrophages

**DOI:** 10.1186/s12885-023-10733-5

**Published:** 2023-03-20

**Authors:** Xin Liu, Zhenke Li, Jinxing Sun, Zhijie Zhang, Weiguo Li

**Affiliations:** 1 Department of Ultrasound, Qilu Hospital of Shandong University, No. 107 Wenhua West Road, Jinan, Shandong 250012 P.R. China; 2 Department of Neurosurgery, Qilu Hospital of Shandong University, No. 107 Wenhua West Road, Jinan, Shandong 250012 P.R. China; 3 Department of Ultrasound, Shandong Maternal and Child Health Hospital, No.238 Jingshi East Road, Jinan, Shandong 250014 P.R. China

**Keywords:** Glioblastoma, Tumor-associated macrophages, PD-L1, sVEGFR1

## Abstract

**Purpose:**

The combined application of immune checkpoint inhibitors (ICIs) and anti-angiogenesis therapy has shown synergistic effects on glioblastoma (GBM). As important resources of PD-L1 in the tumor microenvironment (TME), tumor-associated macrophages (TAMs) have significant impact of the efficiency of ICIs. However, the effects of anti-angiogenesis agents on immune checkpoints expression are not fully understood.

**Method:**

GBM-educated macrophages were generated from circulating monocytes of healthy controls and GBM patients under the education of GBM cell line. Surface expression of PD-L1 and VEGFR1 on GBM-educated macrophages was analyzed. VEGFR1 NAb and soluble VEGFR1 (sVEGFR1) were added and their effects on PD-L1 expression on TAMs was investigated. Serum soluble PD-L1 (sPD-L1) and sVEGFR1 levels in GBM patients were measured and their correlation was analyzed.

**Result:**

The expression intensity of PD-L1 on GBM-educated macrophages was higher and its up-regulation partially depends on VEGFR1 signaling pathway. GBM-educated macrophages secreted less levels of soluble VEGFR1 (sVEGFR1), and exogenous sVEGFR1 down-regulated PD-L1 expression intensity. PD-L1 blockade promoted the secretion of sVEGFR1. Finally, sVEGFR1 and sPD-L1 in serum of GBM patients were overexpressed, and a positive correlation was found.

**Conclusion:**

These findings reveal the interaction between PD-L1 and VEGFR1 signaling pathway in GBM-educated macrophages. VEGFR1 is involved with PD-L1 overexpression, which can be impeded by autocrine regulation of sVEGFR1. sVEGFR1 secretion by GBM-educated macrophages can be promoted by PD-L1 blockade. Taken together, these findings provide evidences for the combined application of ICIs and anti-angiogenesis therapies in the treatment of GBM.

**Supplementary Information:**

The online version contains supplementary material available at 10.1186/s12885-023-10733-5.

## Introduction

Glioblastoma (GBM) is the most common malignant tumor of the central nervous system with 5-year survival rate of approximate 10% [1]. A new therapeutic strategy is urgently needed to overcome the limitations of conventional treatment and improve the prognosis of patients. Immune checkpoint inhibitors (ICIs) are one of the most promising therapeutic approaches. However, unlike the outstanding effects on some types of tumors such as melanoma, results of clinical trials by now have shown that ICIs have limited effects on GBM [2, 3]. The immunosuppressive tumor microenvironment (TME) of GBM is one of the limiting factors of treatment efficiency of ICIs. Macrophages are an important part in the TME, accounting for 80% of all immune cells [4]. Tumor associated macrophages (TAMs) express significant higher level of PD-L1 than other immune cells in both tumor and stromal compartment [5]. Overexpression of PD-L1 in TAMs impairs their activity and proliferation, and results in immunosuppressive phenotypes [6]. Therefore, PD-L1 on TAMs affects antitumor immune activity in TME, and might be a potential therapeutic target. However, the factors affecting PD-L1 expression on TAMs have not been fully elucidated.

Compared with ICIs, anti-angiogenesis therapy has been applied in GBM treatment for longer time and has become a mature complementary treatments for advanced GBM. Clinical trials in a variety of solid tumors have confirmed that the combined application of ICIs and anti-angiogenesis therapy shows stronger therapeutic effect than monotherapy [7]. In GBM-bearing mice, ICIs such as anti-PD-L1 blockade improves the efficacy of anti-VEGF and markedly extends survival benefit [8]. Anti-angiogenesis therapy may improve the local immunogenicity of tumor, so as to enhance the therapeutic effect of ICIs, from the following aspects: First, inhibition of abnormal angiogenesis reduces the accumulation of immune-suppressive T cells (Tregs) and promotes the infiltration of tumor-killing T effector cells [9]; Second, inhibiting neo-vasculatures improves hypoxia status in TME and reduce the secretion of immunosuppressive factors such as IDO, IL-6 and IL-10 [10]; Third, inhibiting neo-vasculatures reduces the inhibitory effect of VEGF on the maturation of myeloid immune cells such as dendritic cells and macrophages [11]. In addition, anti-VEGF antibody promotes the expression intensity of immune checkpoints such as PD-1, CTLA-4 and TIM-3 on CD8^+^ T cells [12]. This finding suggests that anti-VEGF can directly regulate the expression of immune checkpoints on immune cells and therefore affect the therapeutic effect of ICIs. However, whether there is a similar effect on TAMs remains largely unknown.

In the present study, we explored the interaction between PD-L1 and VEGFR signaling on GBM-educated macrophages. The results showed that the expression intensity of PD-L1 on GBM monocyte-derived macrophages was higher than that of healthy control monocyte-derived macrophages. The up-regulation of PD-L1 expression on TAMs is regulated by IFN-γ and partially depends on the activity of VEGFR1 signaling pathway. The level of soluble VEGFR1 (sVEGFR1) secreted by TAMs derived from GBM monocytes is lower, so the abnormal over-activation of VEGFR1 signal pathway may be an important factor for the up-regulation of PD-L1 expression. PD-L1 blockade promoted the secretion of sVEGFR1 from GBM-educated macrophages. Finally, the levels of sVEGFR1 and sPD-L1 in serum of GBM patients were significantly up-regulated, which was positively correlated with tumor grades. Our results provide theoretical evidences for the combined application of ICIs and anti-angiogenesis therapy for glioblastoma by regulating phenotypes and functions of TAMs.

## Materials and methods

### Collection of human samples

This study was conducted in accordance with the Declaration of Helsinki and was approved by the Human Research Ethics Committee of the Qilu Hospital of Shandong University (China). Consent was achieved before sample collection and subsequent analyses. Patients with newly diagnosed GBM, who had not received radiotherapy, chemotherapy or other types of antitumor treatment, and had not previously been diagnosed with other types of tumors, were recruited. Peripheral blood samples were collected from the GBM patients before surgery and from healthy volunteers and stored in K_2_EDTA- or coagulant-containing BD vacutainers (United Kingdom).

### Isolation of human peripheral blood monocytes and T cells

For the isolation of peripheral blood monocytes, PBMCs were obtained from peripheral blood of healthy volunteers or GBM patients by centrifugation with Ficoll-Paque Plus (Sigma-Aldrich). CD14^+^ monocytes and CD3^+^ T cells were isolated from PBMCs by positive selection using anti-CD14- or anti-CD3-conjugated magnetic microbeads respectively (Miltenyi Biotech), according to the manufacturer’s instructions. The purity was above 95%, as determined by flow cytometry.

### Cell culture and generation of GBM-educated macrophages

The human GBM cell line U87 and mouse microglia cell line BV-2 were purchased from Procell Life Science&Technology (China). The mouse GBM cell line GL261 was purchased from OriCell (China). The human GBM cell line U87 was cultured in RPMI 1640 medium supplemented with 10% FBS, 100 U/mL penicillin, and 100 mg/mL streptomycin at 37 °C in an incubator with 5% CO_2_. In order to obtain conditioned medium (CM), 2 × 10^5^/mL U87 cells were seeded into six-well plate for 48 h, and the supernatant was collected. The mouse GBM cell line GL261 and mouse microglia cell line BV-2 were cultured in DMEM with 10% FBS, 100 U/mL penicillin, and 100 mg/mL streptomycin. The CM of GL261 was collected with the same protocol as U87 cells. To generalize GBM-educated microglia, BV-2 cells were cultured in mixture of fresh DMEM complete medium and GL261 CM with a ratio of 1:1 for 48 h.

To generalize monocyte-derived macrophages and polarize them into M1 or M2 macrophages in vitro, CD14^+^ primary monocytes were cultured with rhM-CSF (50 ng/mL, R&D) in RPMI-1640 complete medium for seven days, supplemented with M1-polarizing factors (LPS 100 ng/mL and rhIFN-γ 1 ng/mL) or M2-polarizing factors (IL-10 and IL-4, both 10 ng/mL). Expression of surface markers, such as CD80 and CD206, on M0, M1 and M2 cells were shown as Supplemental Fig. 1.

**Fig. 1:**
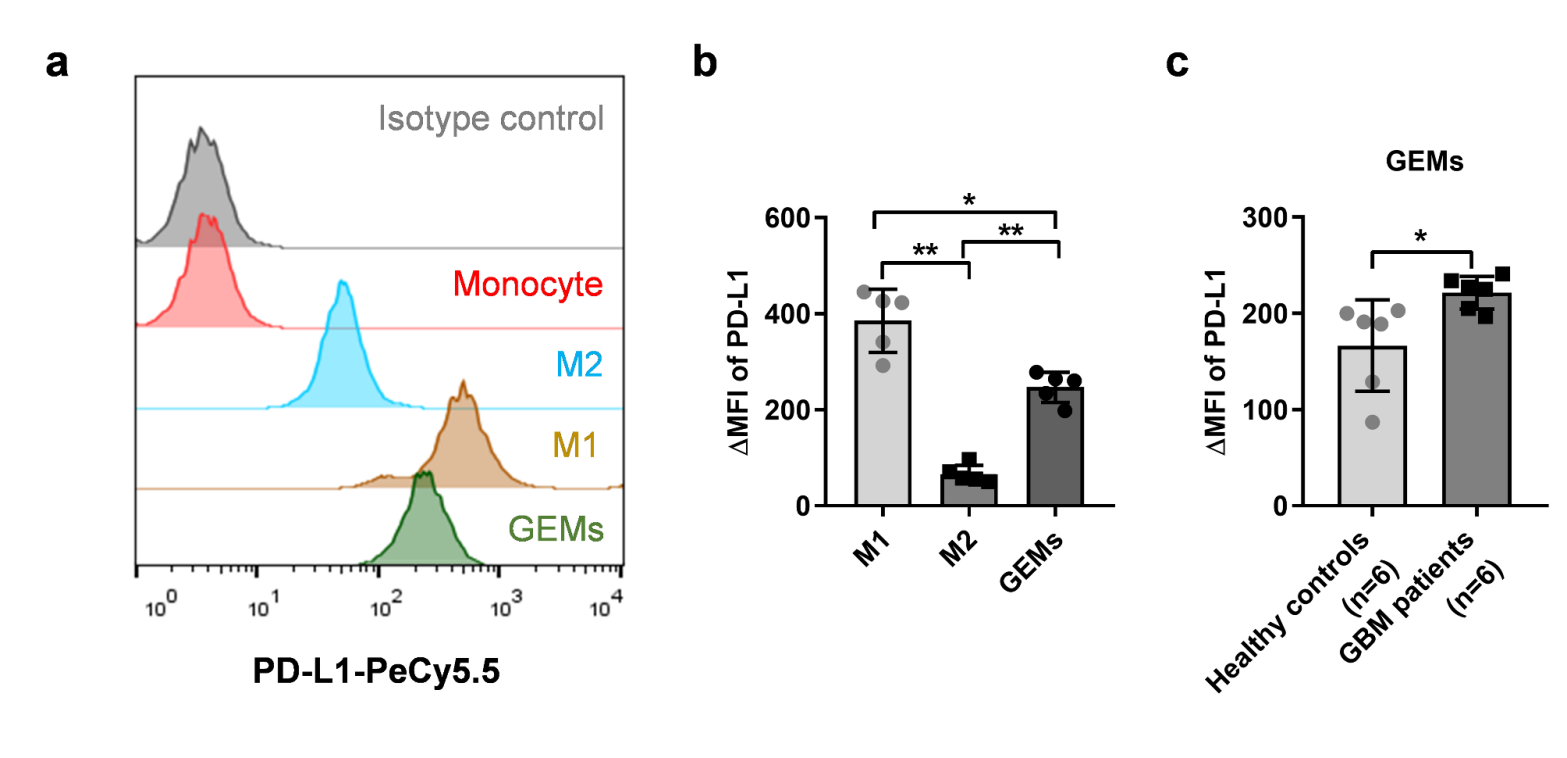
**The expression intensity of PD-L1 on monocytes and macrophages. (a)** Typical flow-cytometry results of PD-L1 surface expression on circulating monocytes from a healthy control, or their derived M1, M2 or GBM-educated macrophages. **(b)** ΔMFI values of PD-L1 on M1, M2 or GBM-educated macrophages, derived from circulating monocytes of healthy controls (n = 5). * *P* < 0.05, ** *P* < 0.01. **(c)** ΔMFI values of PD-L1 on GBM-educated macrophages, derived from circulating monocytes of healthy controls (n = 6), or GBM patients (n = 6). * *P* < 0.05. *P* values are achieved by t-test. GEMs, GBM-educated macrophages

To generalize GBM-educated macrophages, 5 × 10^5^/mL CD14^+^ monocytes were cultured in mixture of RPMI-1640 complete medium and U87 CM with a ratio of 1:1 and rhM-CSF (50 ng/mL). The CM and cytokines were refreshed every other day. After 7 days of culture, the GBM-educated macrophages were harvested for the subsequent analysis. In some experiments, anti-VEGFR1 neutralizing antibody (NAb) (25 µg/mL, Novus Biologicals, Cat: AF321), anti-IFN-γ NAb (10 µg/mL, eBioscience, Cat: 16-7318-81), sVEGFR1 (100 ng/mL, Biolegend, Cat: 555,802) and/or anti-PD-L1 (2 µg/mL, eBioscience, Cat: 16-5983-82) was added at the beginning of differentiation process.

For the co-culture between GBM-educated macrophages and syngeneic T cells, isolated CD3^+^ T cells from peripheral blood was seeded into a Transwell chamber (0.4 μm, Corning) and inserted into six-well plate containing CD14^+^ monocytes from the same person with a ratio of 5:1 (T:M). GBM-educated macrophages was induced according to the protocol as described above.

For functional assay of GBM-educated macrophages on T cells, GBM-educated macrophages was firstly differentiated as the above protocol, and co-cultured with allogeneic CD3^+^ T lymphocytes with a ratio of 5:1 (T:M) in RPMI-1640 complete medium with rhM-CSF (50 ng/mL) and rhIL-2 (20 U/mL) for 5 days. After 5 days, the co-culture conditioned medium was harvested and assessed for IFN-γ production by commercial ELISA kit.

### Flow cytometry

Circulating monocytes or their derived macrophages were stained with anti-PD-L1-PeCy5.5 (Biolegend, Cat: 329,738) or anti-VEGFR1-FITC (Novus Biologicals, Cat: NB100-664 F) antibody. Mouse microglia cell line BV-2 or GBM-educated ones were stained with anti-mouse PD-L1-PE (Biolegend, Cat: 153,612) or anti-mouse VEGFR1-PE (Bio-Techne, Cat: FAB4711P) antibody. Isotype controls were analyzed in parallel. The samples were acquired on a FACSCalibur flow cytometer (BD Biosciences) and analyzed using the FlowJo software. To analyze the expression intensity of PD-L1, ΔMFI values were shown, calculated as the subtracted MFI value between each group and the corresponding isotype controls.

### Enzymes linked immunosorbent assay (ELISA)

Human IFN-γ (Elabscience, China, Cat: E-EL-H0108c), human VEGF (R&D, Cat: DVE00), human sVEGFR1 (R&D, Cat: DVR100C), mouse sVEGFR1 (R&D, Cat: MVR100) and human sPD-L1 (Abcam, Cat: ab277712) levels in serum or cell culture supernatants were quantified using the commercial ELISA kit according to the manufacturers’ instructions.

### Quantitative RT-PCR

RNA from macrophages was extracted using the TRIzol Reagent (Invitrogen). cDNA was synthesised using reverse transcription. Quantitative RT-PCR (qRT-PCR) was conducted on a LightCycler 2.0 Instrument (Roche). GAPDH was used as an internal control. The primer sequences were as follows: sVEGFR1, forward: 5’-AGC ACG CTG TTT ATT GAA AGA GT-3’, reverse: 5’-CCA GAT TAG ACT TGT CCG AGG TT-3’; GAPDH, forward: 5’-TCG GAG TCA ACG GAT TTG GTC GTA-3’, reverse: 5’-CTT CCT GAG TAC TGG TGT CAG GTA-3’.

### Statistical analysis

Experiments were conducted at least thrice, and the data are presented as the mean ± SD. Student’s t-test were used for statistical comparisons. The correlation between two variables was determined using Pearson correlation. All statistical analyses were conducted using the SPSS software version 13.0. Statistical significance was set at *P* < 0.05.

## Results

### Macrophages derived from monocytes of GBM patients express higher level of PD-L1 compared with those derived from monocytes of healthy controls

First, CD14^+^ monocytes were isolated from peripheral blood and differentiated into macrophages under the education of conditioned medium (CM) of GBM cell line U87. The expression level of PD-L1 on GBM-educated macrophages was detected by flow cytometry. As shown in Fig. 1A, monocytes hardly expressed PD-L1, but M1, M2 or GBM-educated macrophages strongly expressed PD-L1. Although the positive rates were greater than 95% in all three types of macrophages, the expression intensity of PD-L1, represented as mean fluorescence intensity (MFI), on M1 and GBM-educated macrophages was higher than that of M2 macrophages (Fig. 1B). The mouse microglia cell line BV-2 expressed low level of PD-L1, with a positive rate of approximate 10%, and up-regulated to approximate 20% in GBM-educated BV-2 cells (Supplemental Fig. 2A and 2B).

**Fig. 2:**
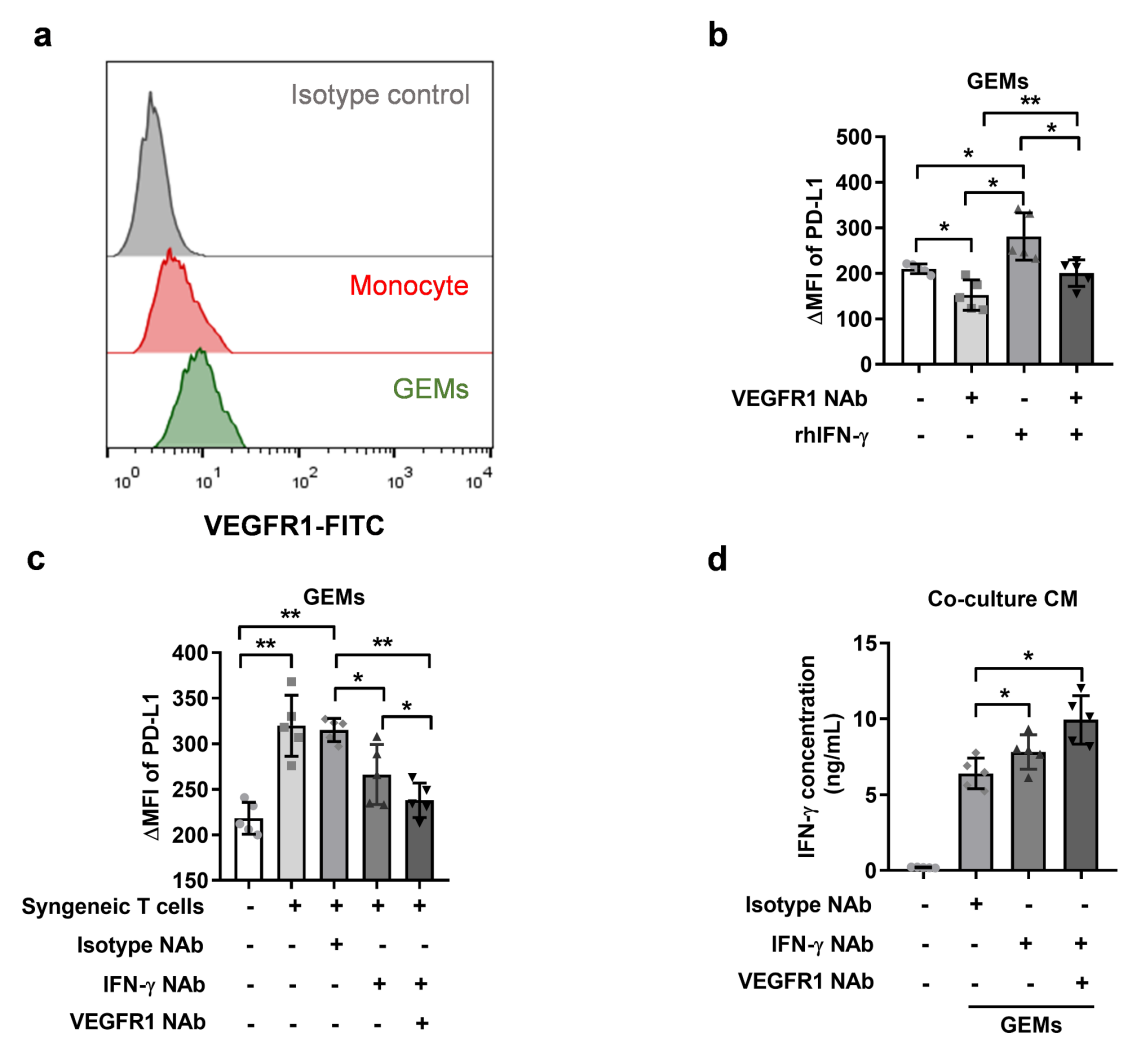
**Blockage VEGFR1 counteracts IFN-γ-mediated PD-L1 upregulation in monocyte-derived macrophages. (a)** Typical flow-cytometry results of VEGFR1 surface expression on circulating monocytes from a healthy volunteer or their derived macrophages (n = 5). **(b)** ΔMFI values of PD-L1 on GBM-educated macrophages (n = 5), treated with VEGFR1 NAb and/or rhIFN-γ. * *P* < 0.05, ** *P* < 0.01. **(c)** ΔMFI values of PD-L1 on GBM-educated macrophages (n = 5), co-cultured with syngeneic T cells, and treated with VEGFR1 NAb and/or IFN-γ NAb. * *P* < 0.05, ** *P* < 0.01. **(d)** IFN-γ concentration in the co-culture supernatant containing CD3^+^ T cells and allogeneic GBM-educated macrophages, pre-treated by VEGFR1 NAb and/or IFN-γ NAb (n = 5). * *P* < 0.05. *P* values are achieved by t-test. GEMs, GBM-educated macrophages

Next, we isolated monocytes from the peripheral blood of GBM patients or healthy volunteers, induced them to differentiate into GBM-educated macrophages in vitro, and detected the expression intensity of PD-L1. Interestingly, we found that the MFI value of PD-L1 on GBM monocyte-derived macrophages was significantly increased compared with that on healthy control monocyte-derived macrophages (Fig. 1C).

### Blockage VEGFR1 counteracts IFN-γ-mediated PD-L1 upregulation in GBM-educated macrophages

We continue to explore the factors that may lead to the over-expression of PD-L1 on GBM monocyte-derived macrophages. We found that the expression of VEGFR1 was up-regulated on GBM-educated macrophages compared with monocytes (Fig. 2A). VEGFR1 was also found on BV-2 cells and GBM-educated BV-2 cells (Supplemental Fig. 2C). The supplementation of VEGFR1 NAb during the differentiation process inhibited the expression intensity of PD-L1 on GBM-educated macrophages (Fig. 2B).

IFN-γ is recognized as inducer of immune checkpoint molecules [13]. Therefore, we added rhIFN-γ during differentiation with/without VEGFR1 NAb and found that IFN-γ enhanced the expression intensity of PD-L1 on GBM-educated macrophages (Fig. 2B). Notably, VEGFR1 blockade counteracted the promoting effect of IFN-γ on PD-L1 expression (Fig. 2B). IFN-γ also promoted PD-L1 expression on GBM-educated BV-2 cells, and VEGFR1 blockade reversed the promoting effect of IFN-γ in GBM-educated BV-2 cells (Supplemental Fig. 2D). Considering that in the tumor microenvironment, T cells are the main resource of IFN- γ, we isolated circulating CD3^+^ T lymphocytes from healthy controls for indirect co-culture with syngeneic GBM-educated macrophages during the differentiation process. With the co-culturing of T cells, the expression intensity of PD-L1 on GBM-educated macrophages was significantly enhanced. Anti-IFN-γ NAb, however, decreased PD-L1 expression intensity on GBM-educated macrophages, and anti-VEGFR1 NAb showed synergistic effect with anti-IFN-γ NAb (Fig. 2C).

For the functional assay of GBM-educated macrophages, we co-cultured GBM-educated macrophages with allogeneic T cells and analyzed IFN-γ concentration in the CM. As Fig. 2D showed, GBM-educated macrophages treated with anti-IFN-γ NAb caused IFN-γ up-regulation in the co-culture CM compared with those treated with isotype control NAb. Moreover, GBM-educated macrophages treated with combined anti-VEGFR1 and anti-IFN-γ NAbs further promoted IFN-γ expression than those treated with anti-IFN-γ NAb alone. However, no significance was reached.

We also analyzed VEGF secretion by monocytes and GBM-educated macrophages. As shown by Supplementary Fig. 3A, monocytes produced low level of VEGF, whereas GBM-educated macrophages produced relatively large amount of VEGF. When indirectly co-cultured with syngeneic T cells, the VEGF production was significantly up-regulated. Adding IFN-γ promoted VEGF production by GBM-educated macrophages (Supplementary Fig. 3B). In the indirect co-culture system between syngeneic T cells and GBM-educated macrophages, IFN-γ NAb significantly decreased VEGF level in the conditioned medium. The combination of IFN-γ and VEGFR NAb further slightly inhibited VEGF production, but no significance was reached compared with IFN-γ NAb alone (Supplementary Fig. 3C).

**Fig. 3:**
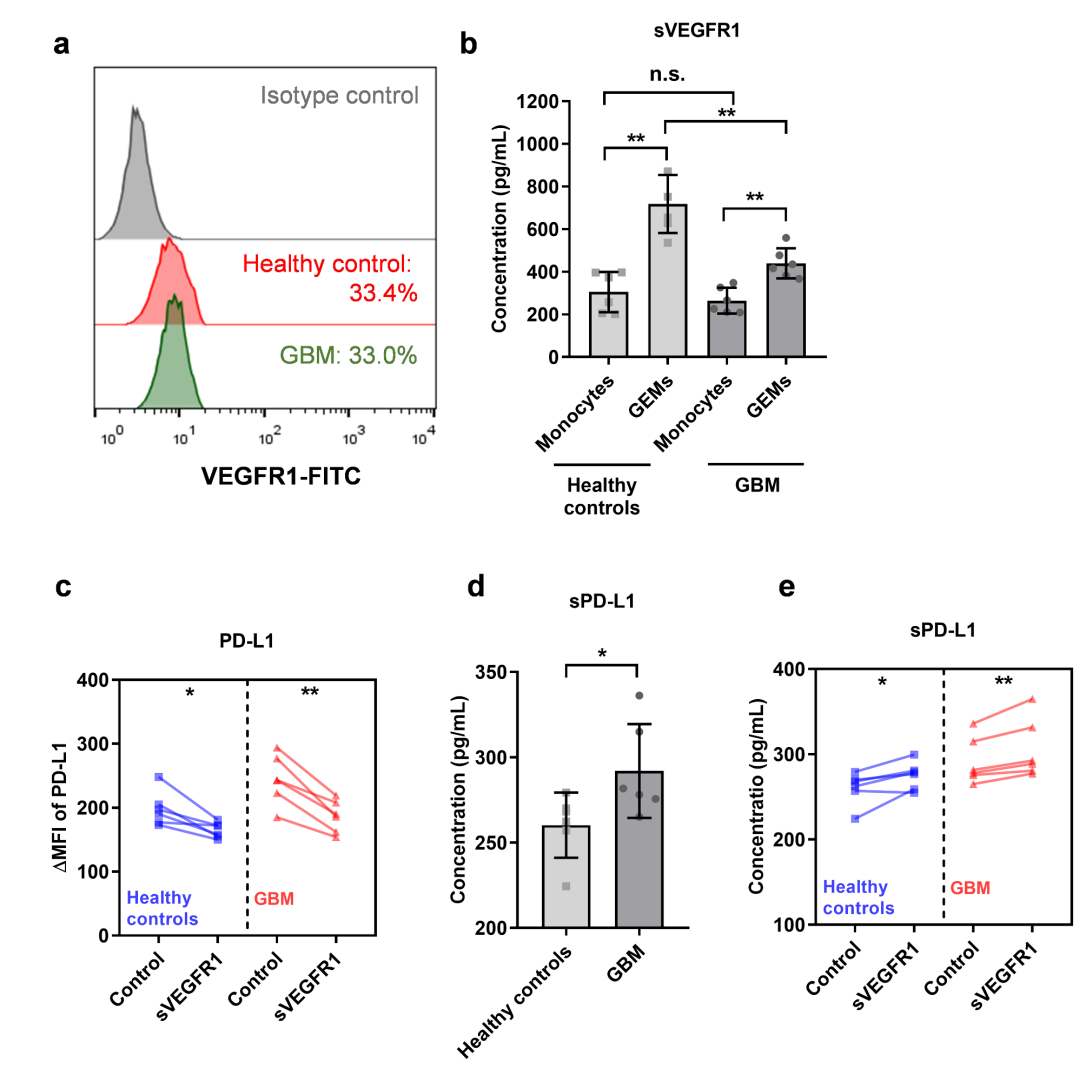
**sVEGFR1 down-regulates PD-L1 but promoted sPD-L1 expression on GBM-educated macrophages. (a)** Typical flow-cytometry results of VEGFR1 surface expression on macrophages derived from circulating monocytes of a healthy control or a GBM patient. **(b)** sVEGFR1 levels in the supernatant of monocytes or their derived macrophages from healthy controls (n = 6) and GBM patients (n = 6). ** *P* < 0.01. n.s. not significant. **(c)** PD-L1 on healthy control or GBM monocyte-derived macrophages (both n = 6), treated with sVEGFR1. * *P* < 0.05, ** *P* < 0.01, control vs. sVEGFR1. **(d)** sPD-L1 levels in the supernatant of healthy control or GBM monocyte-derived macrophages (both n = 6), activated by LPS (100ng/mL). * *P* < 0.05. **(e)** sPD-L1 levels in the supernatant of healthy control or GBM monocyte-derived macrophages (both n = 6), treated with sVEGFR1 and LPS. * *P* < 0.05, ** *P* < 0.01, control vs. sVEGFR1. *P* values are achieved by t-test. GEMs, GBM-educated macrophages

### Lower secretion of sVEGFR1 in GBM monocytes-derived macrophages is associated with differential PD-L1 and soluble PD-L1 expression

The above results suggest that VEGFR1 is an important regulatory factor of PD-L1 expression on GBM-educated macrophages. Therefore, we detected the expression level of VEGFR1 on GBM monocyte- and healthy control monocyte-derived macrophages, respectively. However, there was no significant difference in VEGFR1 expression between these two groups (Fig. 3A), so we further analyzed whether there were other factors. sVEGFR1 regulates the activity of VEGFR signaling by competitive binding with VEGF. Therefore, we detected the secretion level of sVEGFR1 in two groups of monocytes and their-derived macrophages. As shown in Fig. 3B, there was no significant difference in the secretory level of sVEGFR1 between GBM and healthy control monocytes. However, after their differentiation into GBM-educated macrophages, the secretory level of sVEGFR1 of GBM monocyte-derived macrophages was lower than that of healthy control monocyte-derived macrophages (Fig. 3B). Similarly, BV-2 cells were also found to secret sVEGFR1, and the secretion level was further up-regulated after they were educated by GBM cells (Supplemental Fig. 2E).

In view of the above findings, we added exogenous human sVEGFR1 during the differentiation of GBM-educated macrophages, and found that exogenous sVEGFR1 induced down-regulation the expression intensity of PD-L1 on healthy control or GBM monocyte-derived macrophages (Fig. 3C).

Soluble PD-L1 (sPD-L1) was the trans-acting extracellular form of PD-L1, which was secreted by a variety of activated immunocytes and malignancies. We analyzed sPD-L1 levels in the supernatant of LPS-activated macrophages derived from monocytes of healthy controls or GBM patients, and found that GBM monocyte-derived macrophages produced higher level of sPD-L1 (Fig. 3D). Adding exogenous sVEGFR1 enhanced sPD-L1 production in both healthy control and GBM monocyte-derived macrophages (Fig. 3E).

### PD-L1 blockade increases production of sVEGFR1 in GBM-educated macrophages

**Fig. 4:**
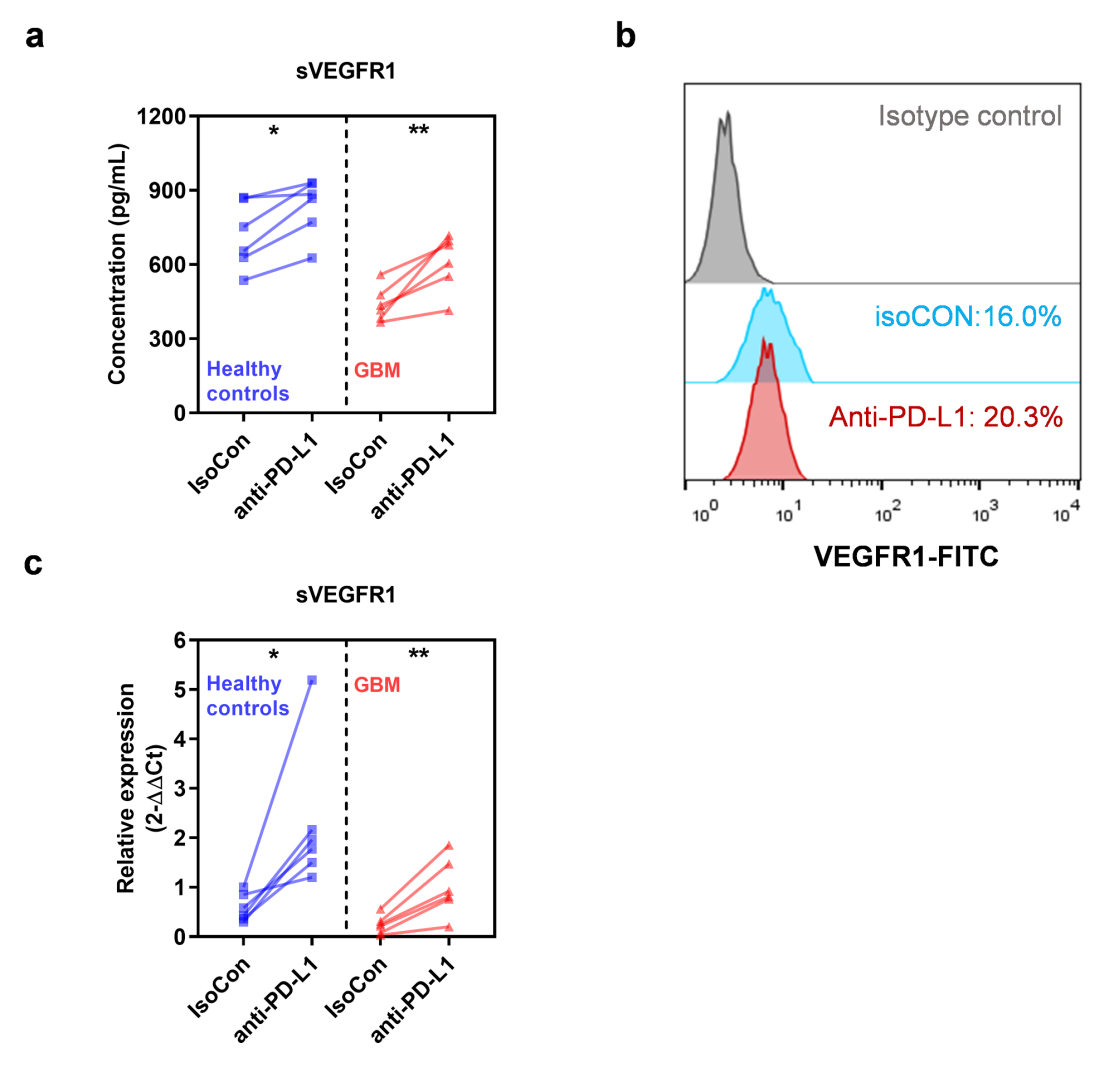
**PD-L1 blockade increases production of sVEGFR1 from GBM-educated macrophages. (a)** sVEGFR1 levels on the supernatant of healthy control or GBM monocyte-derived macrophages (both n = 6), treated with isotype control or anti-PD-L1. * *P* < 0.05, ** *P* < 0.01, isotype control vs. anti-PD-L1. **(b)** Typical flow-cytometry results of VEGFR1 surface expression on GBM-educated macrophages, treated with isotype control or anti-PD-L1. **(c)** Relative sVEGFR1 mRNA levels of in healthy control or GBM monocyte-derived macrophages (both n = 6), treated with isotype control or anti-PD-L1. * *P* < 0.05, ** *P* < 0.01, isotype control vs. anti-PD-L1. *P* values are achieved by t-test

Immune checkpoint inhibitors and anti-angiogenesis therapies are considered to have synergistic effects in tumor treatment [7]. Therefore, we continue to explore whether PD-L1 blockade affects VEGF signaling pathway. We found that after the addition of anti-PD-L1, the secretory level of sVEGFR1 of both healthy control monocyte and GBM monocyte-derived macrophages increased (Fig. 4A). PD-L1 blockade had no significant effect on the expression level of VEGFR1 on the surface of GBM-educated macrophages (Fig. 4B), but significantly promoted the expression level of sVEGFR1 mRNA (Fig. 4C), suggesting that PD-L1 blockade may regulate its secretion by affecting the *de novo* synthesis of sVEGFR1.

### Serum PD-L1 and sVEGFR1 levels of GBM patients are associated with tumor grades

**Fig. 5:**
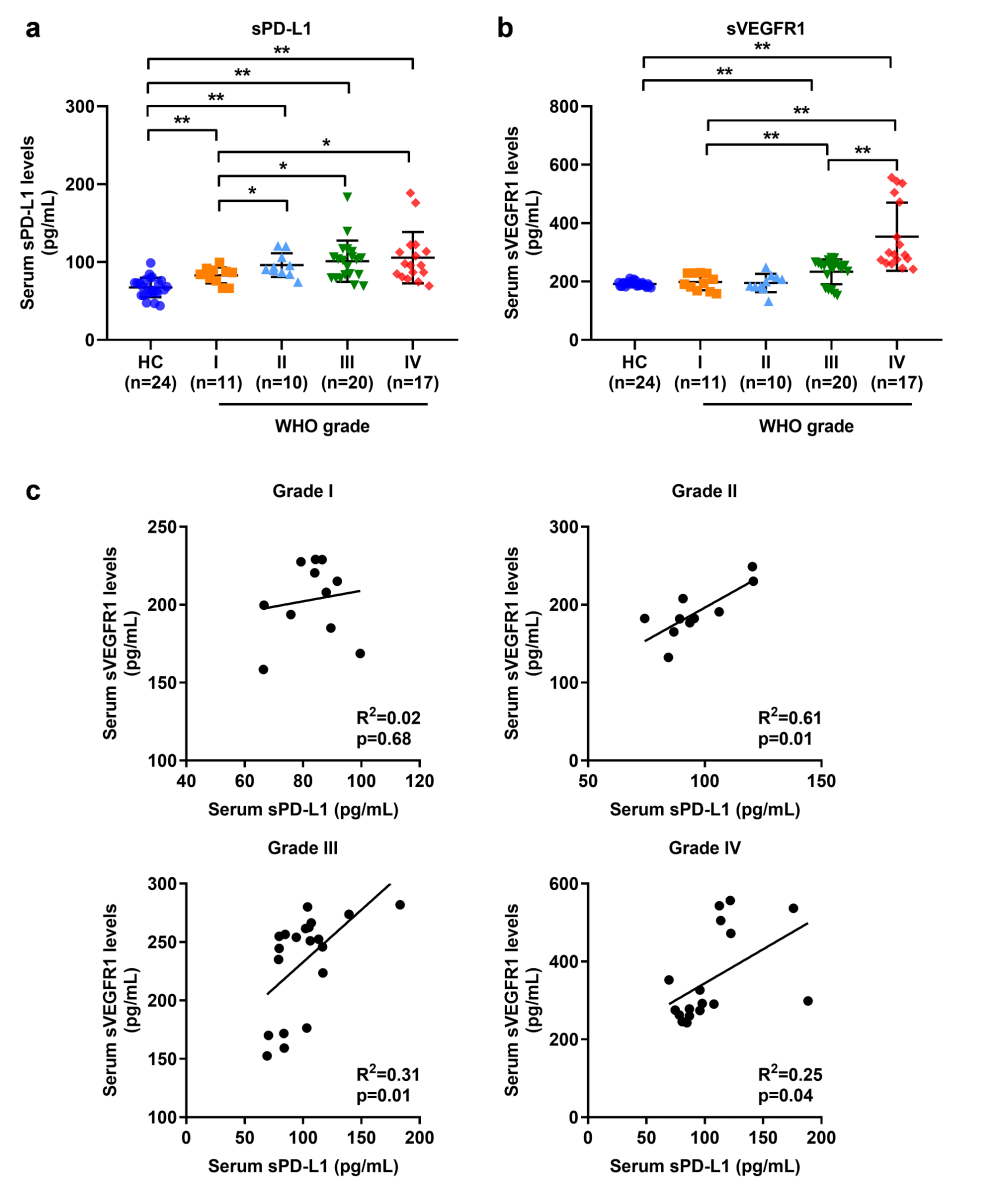
**Correlation of serum sPD-L1 and sVEGFR1 in GBM patients. (a)** Serum sPD-L1 levels in healthy controls (HC, n = 24) and GBM patients (WHO grade I, n = 11; grade II, n = 10; grade III, n = 20; grade IV, n = 17). * *P* < 0.05, ** *P* < 0.01. *P* values are achieved by t-test. **(b)** Serum sVEGFR1 levels in healthy controls (HC, n = 24) and GBM patients (WHO grade I, n = 11; grade II, n = 10; grade III, n = 20; grade IV, n = 17). ** *P* < 0.01. *P* values are achieved by t-test. **(c)** Correlation between serum sPD-L1 and sVEGFR1 in GBM patients with TNM grade I (n = 11), grade II (n = 10), stage III (n = 20) or stage IV (n = 17). R^2^ and *P* values are achieved by Pearson correlation test

Finally, we detected the levels of sPD-L1 and sVEGFR1 in the serum of GBM patients and found that their levels were higher than those in healthy controls (Fig. 5A and B). Interestingly, the levels of serum sPD-L1 and sVEGFR1 in GBM patients with WHO grade IV were significantly higher than those in patients with WHO grade I (Fig. 5A; Table 1). In GBM patients with grade II, III and IV, the levels of serum sPD-L1 and sVEGFR1 were positively correlated. However, in those with grade I, no significant correlation between sPD-L1 and sVEGFR1 was observed (Fig. 5C).

**Table 1:** Serum sPD-L1 and sVEGFR1 values of GBM patients with different clinicopathological parameters

	sPD-L1 (mean ± SD, pg/mL)	sVEGFR1 (mean ± SD, pg/mL)
**Gender**		
Male (n = 29)	90.60 ± 15.57	279.87 ± 96.39
Female (n = 29)	105.66 ± 31.36	232.16 ± 89.32☆☆
**Age**		
< 65 (n = 33)	94.93 ± 23.05	263.34 ± 105.40
≥ 65 (n = 25)	102.40 ± 28.74	245.36 ± 80.40
**WHO grade**		
I (n = 11)	82.91 ± 10.22	198.46 ± 27.87
II (n = 10)	96.23 ± 15.26★	195.14 ± 31.29
III (n = 20)	101.10 ± 26.41★	233.70 ± 42.60★★
IV (n = 17)	105.62 ± 33.09★	353.80 ± 116.80★★
**Karnofsky Performance Scale**		
< 80 (n = 33)	100.94 ± 26.71	239.96 ± 83.37
≥ 80 (n = 25)	94.42 ± 24.32	276.24 ± 106.94

☆☆ *P* < 0.05, male vs. female;

★ *P* < 0.05, ★★ *P* < 0.01, grade II, III or IV vs. grade I

## Discussion

The expression of PD-L1 on TAMs is an important factor leading to tumor immune escape, but the regulatory mechanism affecting PD-L1 expression has not been fully clarified. In this study, we unexpectedly found that the expression level of PD-L1 on macrophages derived from peripheral blood monocytes of GBM patients was significantly higher than that of healthy controls. VEGFR1 was up-regulated during the differentiation of monocytes into GBM-educated macrophages, which led to the up-regulation of IFN-γ-dependent PD-L1 overexpression. Compared with healthy control monocyte-derived macrophages, the secretion level of soluble VEGFR1 (sVEGFR1) in GBM monocyte-derived macrophages decreased significantly, resulting in over-activation of VEGFR1 signaling pathway and PD-L1 overexpression. Supplementation of exogenous sVEGFR1 decreased PD-L1 expression intensity on GBM monocyte-derived macrophages. PD-L1 blockade promoted the secretion of sVEGFR1 in GBM-educated macrophages, thus forming a feedback regulation to further inhibit the expression of PD-L1. Finally, the found that levels of serum sPD-L1 and sVEGFR1 in patients with advanced GBM grade (grade IV) were higher than those in patients with early grade (grade I). There was a positive correlation between the levels of serum sVEGFR1 and sPD-L1 in patients with the same tumor grade. Our study reveals the interaction between PD-L1 and VEGFR signaling pathway in GBM-educated macrophages, which may provide theoretical evidences for the combined application of immune checkpoint inhibitors (ICIs) and anti-angiogenesis therapies in the treatment of GBM.

GBM cells disrupt the integrity of the brain-blood barrier (BBB) and release a variety of monocyte/macrophage chemokines, and therefore promote the recruitment of peripheral monocytes and accumulation of monocyte-derived macrophages at the tumor site [14]. Previous studies have shown that TAMs in GBM are heterogeneous cell populations composed of M1/M2 polarized macrophages [15]. This was in line with our study, as we found that the expression level of M1 (CD80) or M2 (CD206) markers on GBM-educated macrophages induced in vitro was between M1 and M2-polarized macrophages (Supplemental Fig. 1). Moreover, our results showed that the expression intensity of PD-L1 on GBM-educated macrophages was higher than that of M2 macrophages induced in vitro, which was consistent with the results of previous studies [16]. Importantly, we found that GBM monocyte-derived macrophages expressed higher level of PD-L1 than healthy control-derived ones. To our knowledge, this is the first report that peripheral monocytes of GBM patients or healthy donors can affect the expression level of PD-L1 in their differentiated GBM-educated macrophages. The above results suggest that the polarization state and their expression of immune checkpoints receptors (such as PD-L1) of macrophages may not only be affected by the TME, but also be affected by the characteristics of their precursor monocytes. However, how peripheral monocytes decide the phenotypes and functions of TAMs needs further study.

There are few but important studies that confirm the correlation between angiogenesis and PD-L1 expression in tumor tissue. A positive correlation between PD-L1 and VEGF was found in clear cell renal cell carcinoma, and was associated with the poor prognosis of patients [17]. Another in vitro study confirmed that exogenous supplementation of angiopoietin-2 (ANGPT2), which was another proangiogenic cytokines that sustain tumor angiogenesis, promoted the expression of PD-L1 on M2 polarized macrophages [18]. The above studies confirmed that tumor cells or macrophages can receive VEGF signals to promote the expression of PD-L1. Similar crosstalk was observed in GBM, as VEGF pathway inhibitors in combine with the tricyclic antidepressant imipramine unexpectedly reprogramed immunosuppressive TAMs toward immunostimulatory phenotype, including PD-L1 low expression intensity [8]. Consistent with this study, we confirmed that blocking VEGFR inhibits PD-L1 expression on GBM-educated macrophages. Moreover, we tried to explore whether GBM-educated macrophages can autonomously regulate PD-L1 expression through VEGF signaling pathway. We found that the expression intensity of VEGFR1 does not seem to be the main factor affected PD-L1 expression on GBM-educated macrophages, because our results showed that there was no significant difference in the expression levels of VEGFR1 in healthy control monocytes, GBM monocytes or their differentiated macrophages. On contrary, the differential secretion of sVEGFR1 may be an important factor affecting the activity of VEGF signaling pathway. After exogenous sVEGFR1 was supplemented in the process of differentiation, the expression intensity of PD-L1 on TAMs decreased to a level similar to that of healthy control monocyte-derived macrophages. Another important observation is that, PD-L1 blockade significantly promoted the expression of sVEGFR1 in GBM-educated macrophages. As a truncated version of the cell membrane-spanning VEGFR1, sVEGFR1 competitively binds circulating VEGF and inhibits the activity of VEGFR signaling pathway [19]. Therefore, the increased secretion of sVEGFR1 may be a new mechanism by which PD-L1 blockade inhibiting VEGF signaling pathway. Through this crosstalk, a positive feedback regulation was connected between PD-L1 and VEGF pathway on GBM-educated macrophages, which may provide evidences for the synergistic application of anti-PD-1/PD-L1 and anti-VEGFR therapy in GBM treatment.

Finally, a correlation between serum sPD-L1 and sVEGFR1 levels in GBM patients with different tumor grades was also explored. Our study confirmed overexpression of both sPD-L1 and sVEGFR1 in GBM patients and their association with tumor grade. Moreover, there was a positive correlation between sPD-L1 and sVEGFR1 levels in the serum of GBM patients. Higher sPD-L1 levels are associated with worse prognosis of GBM patients [20]. In patients with renal cell carcinoma, higher serum sPD-L1 levels suggest poor reactivity to sunitinib [21]. In line with the above research results, our findings provide additional evidence for the interaction between PD-1/PD-L1 and VEGF/VEGFR signaling pathway. However, the physiological role of these two molecules during GBM development need to be solved. First, the resources of sPD-L1 and sVEGFR1 need to be resolved. Although our results showed that GBM monocyte-derived macrophages produced sPD-L1 upon LPS activation, and sVEGFR1 promoted sPD-L1 production by GBM-educated macrophages, a variety of cell types, including tumor cells, tumor-infiltrated immunocytes and circulating immunocytes are resources of both sPD-L1 and sVEGFR1 [22–25], which could not be excluded. Second, although overexpression sPD-L1 was reported to be associated with poorer ICIs efficiency in some types of tumors [26, 27], no results in GBM have been reported. Statistics on the number or proportion of different types of cells expressing these two molecules in GBM patients will help to confirm whether this correlation is associated with TAMs.

In conclusion, our study confirmed the bidirectional interaction of PD-L1 and VEGFR1 signaling pathway on GBM-educated macrophages, and found that anti-PD-L1 can further inhibit PD-L1 expression through overexpression of sVEGFR1. This study suggests that PD-L1 and VEGFR1 signaling pathways on TAMs may play a synergistic role in the regulation of tumor microenvironment, which is conducive to better understanding of the role of the combination of ICIs and anti-angiogenesis therapies in the regulation of antitumor immunity of GBM.

## Electronic supplementary material

Below is the link to the electronic supplementary material.

Supplementary Material 1

## Data Availability

The datasets generated during and/or analyzed during the current study are available from the corresponding author on reasonable request.
