# Supplementary material for: Interaction between PD-L1 and soluble VEGFR1 in glioblastoma-educated macrophages
Source: BMC Cancer. 2023 Mar 20;23:259. doi: 10.1186/s12885-023-10733-5 (PMC10026501; doi:10.1186/s12885-023-10733-5)
Supplement: Supplementary file 1 — Supplementary Material 1 [file 12885_2023_10733_MOESM1_ESM.pdf]

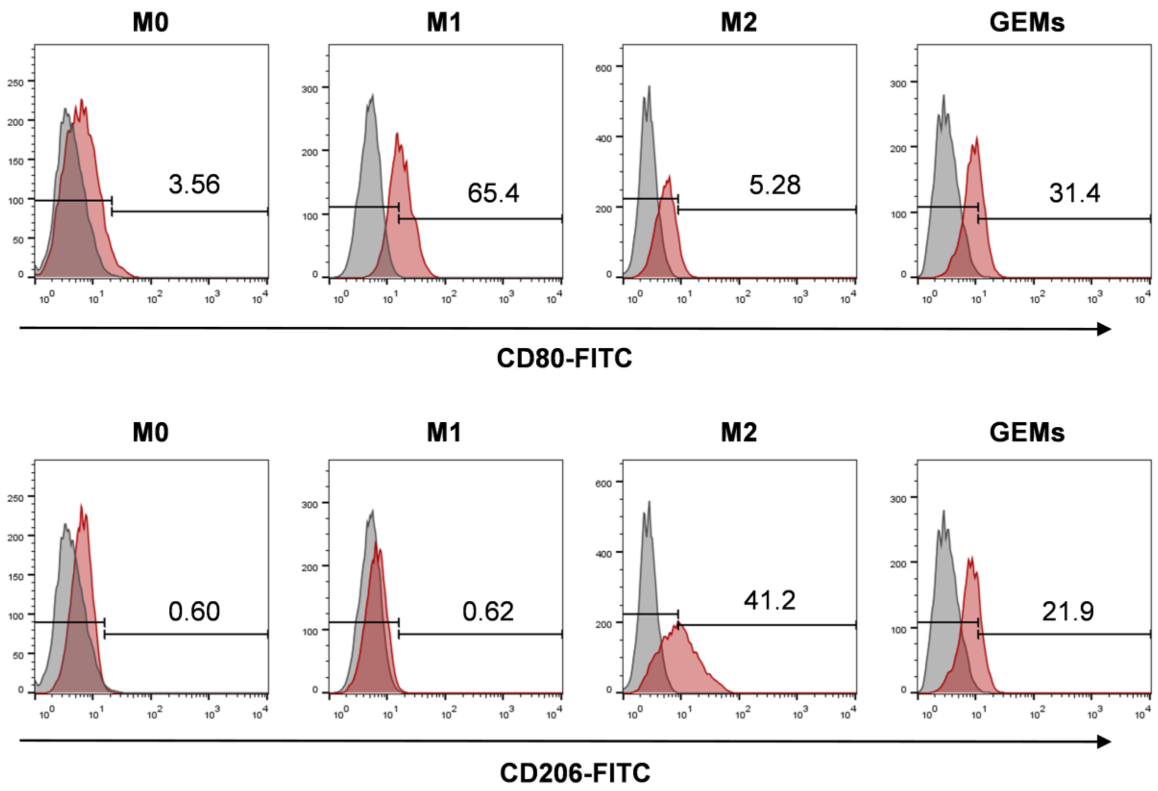

Supplemental Figure 1. The characteristics of monocyte-derived macrophages or GBM-educated macrophages. Typical flow-cytometry results of CD80 (upper) and CD206 (lower) surface expression on monocyte-derived M0, M1, M2 cells in vitro, or GBM-educated macrophages (GEMs).

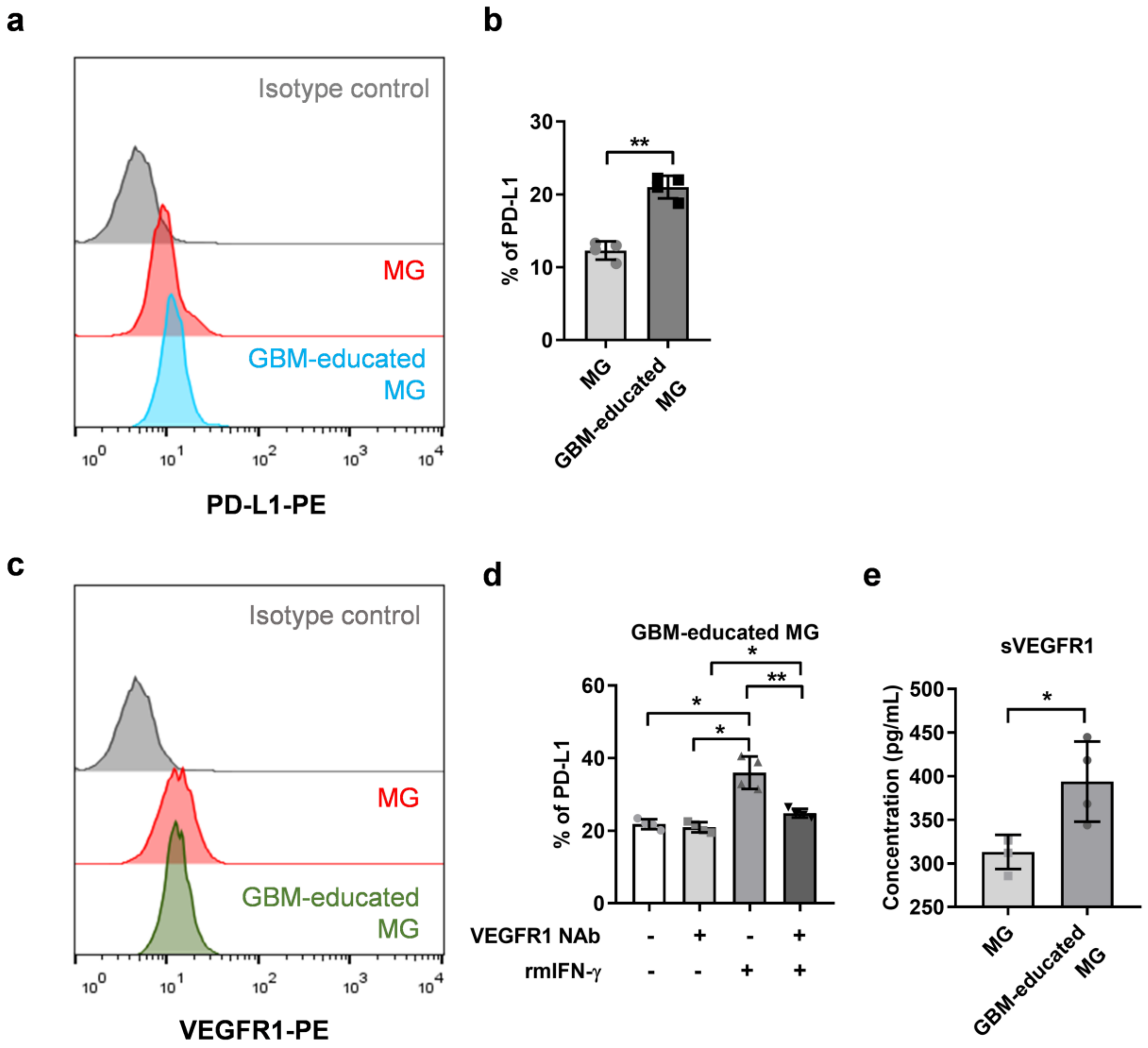

Supplemental Figure 2. The effects of IFN-γ and VEGFR on PD-L1 expression on GBM-educated microglia. a. Typical flow-cytometry results of PD-L1 surface expression on BV-2 cells or GL261-educated BV-2 cells. b. Positive percentage of PD-L1 on the surface of BV-2 cells or GL261-educated BV-2 cells (n=4). ** P<0.01. c. Typical flow-cytometry results of VEGFR1 surface expression on BV-2 cells or GL261-educated BV-2 cells (n=4). d. PD-L1 positive percentage on GL261-educated BV-2 cells (n=4), treated with VEGFR1 NAb and/or rmIFN-γ. * P<0.05, ** P<0.01. e. sVEGFR1 levels in the supernatant of ΔMFI values of BV-2 cells and GL261-educated BV-2 cells (n=4). * P<0.05. P values are achieved by t-test. MG, BV-2 cells; GBM-educated MG, GL261-educated BV-2 cells.

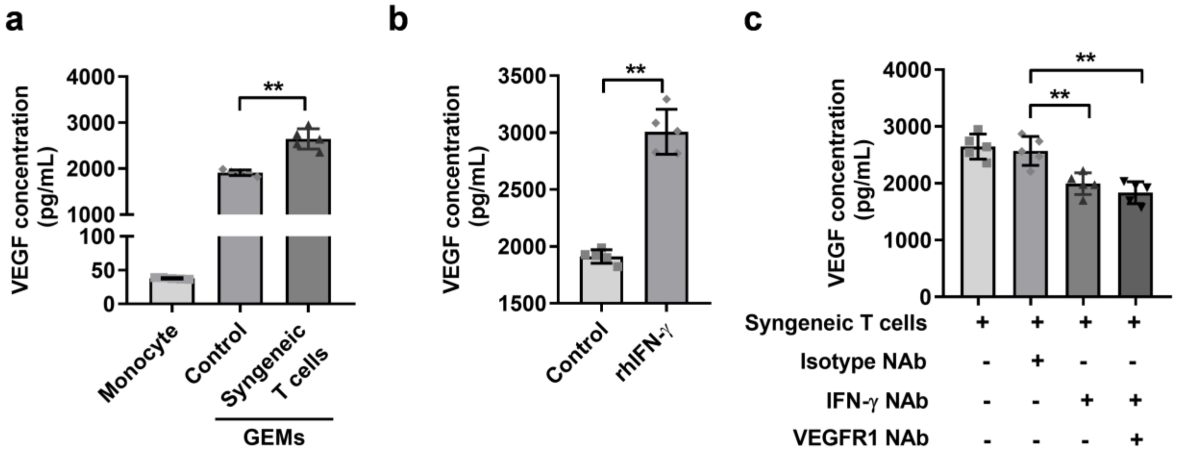

Supplemental Figure 3. The regulation of IFN-γ and VEGFR on VEGF production of GBM-educated macrophages. a. VEGF concentration in the conditioned medium of circulating monocytes, GBM-educated macrophages and macrophages co-cultured with syngeneic T cells (n=5). ** P<0.01. b. VEGF concentration in the conditioned medium of GBM-educated macrophages with or without rhIFN-γ (n=5). * P<0.05. c. VEGF concentration in the conditioned medium of GBM-educated macrophages co-cultured with syngeneic T cells, and treated with VEGFR1 NAb and/or IFN-γ NAb (n=5). * P<0.05. P values are achieved by t-test. GEMs, GBM-educated macrophages.
